# Intraoperative Oxygen Consumption and Postoperative Immune Response in Colorectal Oncological Surgery: A Prospective, Monocentric Pilot Study

**DOI:** 10.3390/jpm14060594

**Published:** 2024-06-01

**Authors:** Robert Ivascu, Madalina Dutu, Sandica Bucurica, Dan Corneci, Cornelia Nitipir

**Affiliations:** 1 Anesthesia and Critical Care Department, Carol Davila University of Medicine and Pharmacy, 020021 Bucharest, Romania; iulian.ivascu@drd.umfcd.ro (R.I.); dan.corneci@umfcd.ro (D.C.); 2 Anesthesia and Critical Care Department, “Dr. Carol Davila” University Emergency Central Military Hospital, 010816 Bucharest, Romania; 3 Gastroenterology Department, Carol Davila University of Medicine and Pharmacy, 020021 Bucharest, Romania; sandica.bucurica@umfcd.ro; 4 Department of Gastroenterology, “Dr. Carol Davila” University Emergency Central Military Hospital, 010816 Bucharest, Romania; 5 Oncology Department, Carol Davila University of Medicine and Pharmacy, 020021 Bucharest, Romania; cornelia.nitipir@umfcd.ro; 6 Oncology Department, Elias University Emergency Hospital, 050474 Bucharest, Romania

**Keywords:** intraoperative oxygen consumption, immune stress response, perioperative neutrophils count dynamic, postoperative surgical outcome

## Abstract

Surgical resection is the key treatment for colorectal cancer, but the extent of surgical trauma has been implied as a key factor for the oncologic outcome. The immune stress response to surgical trauma generates a cascade of immunological events implying neutrophils’ perioperative change generating NETosis, N killer decrease, and platelets’ activation that may influence postoperative surgical outcome, tumor cell growth, and future oncogenesis. The present study aimed to investigate the correlation between intraoperative oxygen consumption (VO_2_) and the dynamic variation of neutrophils, lymphocytes, and platelets in the perioperative period to identify an intraoperative tool that could predict the postoperative immune response. Twenty-six colorectal oncological surgical patients were enrolled in an observational, prospective, monocentric study, over 18 months. Serum neutrophils, lymphocytes, and thrombocytes values were collected in the preoperative period and on the third postoperative day, oxygen consumption was measured and recorded every 15 min during surgery using indirect calorimetry. We compared oxygen consumption measurements registered 30 min after induction of anesthesia (VO_2_a) and the first value registered after abdominal wall closure (VO_2_b) to perioperative variation of absolute neutrophils (VNC), lymphocytes (VLC), and platelets (VPC) count. Our results proved a significant correlation between VO_2_ variation and neutrophils’ perioperative dynamic assessed by VNC (correlation coefficient = 0.547, *p* < 0.01, 95% confidence interval (CI) =0.175, 0.783). We also noticed a correlation between VPC and VO_2_ (correlation coefficient = −0.603, *p* < 0.01, 95% CI = −0.815, −0.248). No correlation could be shown between VO2 and VLC variation (*p* = 0.39). In conclusion, intraoperative VO_2_ variation measured by indirect calorimetry correlates well with perioperative neutrophils and platelets count dynamic variations and can be used as an early prognosis marker of postoperative immune response and surgical outcome in colorectal oncological surgery.

## 1. Introduction

Colorectal cancer is the fourth most common form of cancer, with two million new cases being documented since 2022, and it is estimated that by 2045, the number of newly diagnosed cases will increase above 3.5 million cases. Among newly diagnosed patients, 20% of them have metastatic disease as initial presentation, and another 60% will develop distant relapses. [1,2]. Although surgical resection represents the main treatment for tumoral removal, by direct and indirect mechanisms, surgery may become by itself a predisposing factor for tumor cell growth and future oncogenesis [3,4,5].

The stress response to surgery represents a pattern of physiological and pathophysiological changes that occur in response to surgical trauma, which is influenced by the magnitude, invasiveness, and duration of surgery. The surgical stress response includes two large categories of changes as follows: a neurohormonal response and an immunological response [6].

Tissue injury produces significant local inflammatory changes involving immunological cells’ activation and recruitment, thus disrupting the delicate balance of immunological homeostasis. This period of immune system vulnerability can extend up to a week after surgery [7]. In addition to their roles in defending against various microorganisms and dead cell elimination, neutrophils contribute to the process of metastasis formation through the phenomenon of NETosis [8]. Thus, the immediate postoperative period is characterized by immunosuppression as the number of total lymphocytes, CD8, and N killer cells are decreased, which leads to an increased risk for postoperative complications such as pneumonia, wound infections, and sepsis [9,10]. Also, besides white blood cell activation, platelets activate as a result of the surgical tissue trauma and can become a defensive mechanism for tumor cells by protecting them from N Killer lymphocytes’ action, leading to cancer cell migration and metastatic development [11].

Oxygen is a critical element of energy production, being mainly involved in the oxidative phosphorylation cycle [12]. Oxygen consumption (VO_2_) stands for oxygen taken from the blood and used by the tissues. In normal conditions, it is proportionally related to oxygen delivery (DO_2_) and inversely correlated with cell oxygen extraction. However, oxygen consumption (VO_2_) can be measured by several methods, one of them being indirect calorimetry, which measures inspired and expired gas flows, volumes, and concentrations of O_2_ and CO_2_ in an intubated patient. This direct measurement of oxygen consumption, provided by spirometry, has a much higher accuracy than the indirect methods of calculation based on the reverse Fick equation [13].

Since inadequate surgical stress responses occasionally cause major postoperative complications, intraoperative objective monitors of oxygen consumption for the assessment of surgical stress responses can anticipate postoperative complications.

Considering the impact of surgical trauma on the immune response and the influences of perioperative NETosis, decreasing lymphocytes, and platelets on the oncological outcome, we thought to investigate the relationships between intraoperative oxygen consumption and the dynamic variation of neutrophils, lymphocytes, and platelets during perioperative periods and analyze the link between these relationships and the postoperative immune response. Our aim was to investigate the correlation between intraoperative VO_2_ variation and the dynamics of absolute neutrophil (ANC), absolute lymphocyte (ALC), and absolute platelet count (APC) in the perioperative period to find an intraoperative tool that can predict an earlier immune system’s postoperative evolution. Thus, we compared oxygen consumption variation, measured by indirect calorimetry, with the variation of serum neutrophils, lymphocytes, and platelets.

## 2. Materials and Methods

We conducted an observational, prospective, monocentric study over a period of 18 months, from October 2022 to March 2024, in the Anesthesia and Intensive Care Department of “Carol Davila” Central Military Emergency Hospital, Bucharest, after obtaining approval from the Local Ethics Committee (no. 3272/2 May 2022). All the procedures performed followed the ethical principles of the Declaration of Helsinki, the year 1964 (version 2013), and all patients expressed their written consent.

A total of twenty-six patients were included in the study. Considering that no data were found in the literature about a correlation between intraoperative oxygen consumption and any of the component cells of the immune system, the sample size could not have been estimated before starting the study, so an arbitrary sample size of at least twenty-five patients was used.

The inclusion criteria were adult patients diagnosed with colorectal cancer and scheduled for curative surgical resection. Exclusion criteria consisted of metastatic disease, pregnancy, an associated disease that might interfere with intraoperative measurement monitoring (atrial fibrillation, neurological diseases such as Parkinson’s and Alzheimer’s disease), and surgical techniques that could influence the gas exchange such as laparoscopic or robotic surgery. We also excluded diseases and conditions affecting neutrophils’ perioperative dynamic, such as earlier hematological malignancy, previous chemotherapy, previous blood transfusion, emergency surgery, and infection. Reported allergies to the anesthetics specified in the study’s protocol also represented exclusion criteria.

All patients underwent surgery under general anesthesia, with standard American Society of Anesthesiologists (ASAs) monitoring and advanced nociception (surgical pleth index—SPI) and depth of anesthesia (entropy) monitoring with Carescape B650 Monitor (General Electric Healthcare, Helsinki, Finland). The depth of the neuromuscular block was monitored with a train of four (TOF) (TOFscan Monitor, Drager, Lubeck, Germany).

The induction of general anesthesia was performed with propofol 0.5 mg/kg followed by 0.25 mg/kg boluses at 30 s intervals until the state and response entropy were between 40 and 60, fentanyl 3 micrograms/kg, and lidocaine 2 mg/kg. Rocuronium 0.6 mg/kg was used to facilitate tracheal intubation. Anesthesia was maintained with sevoflurane for an entropy value between 40 and 60, and subsequent boluses of fentanyl and rocuronium were applied following SPI (target values between 20 and 50) and TOF (target value 0 or 1) monitoring. Normothermia was kept by warm liquids administered (3M Ranger Fluid Warming, 3M Company, Saint Paul, MN, USA) in addition to the standard heat conservation technique (warm blankets). All patients were ventilated in volume control mode using a FiO_2_ between 0.4 and 0.5.

Neutrophil, lymphocyte, and platelet count were measured in the preoperative period and on day 3 postoperative by the flow cytometry method using a Sysmex XN 3000 device (Sysmex Corporation, Kobe, Japan). VO_2_ was measured and recorded every 15 min throughout surgery using indirect calorimetry using an E-sCOVX module (General Electric HealthCare, Helsinki, Finland). The module uses side-stream gas sampling and is provided with a paramagnetic sensor for measuring the amount of oxygen. Tidal volume and minute volume ventilation are measured with a pneumotachograph. We noted the following values: VO_2_ 30 min after induction of anesthesia (VO_2_a) and the first VO_2_ value at the end of surgery after abdominal wall closure (VO_2_b).

We used the following mathematical equations to calculate variations of paraclinical results:

Variation of neutrophil count (VNC) = [(ANC on day 3 − preoperative ANC) / preoperative ANC] × 100.

Variation of lymphocyte count (VLC) = [ALC on day 3 − preoperative ALC) / preoperative ALC] × 100.

Variation of platelet count (VPC) = [(APC on day 3 − preoperative APC) / preoperative APC] × 100.

Variation of oxygen consumption (VVO_2_) = [(VO_2_b − VO_2_a) / VO_2_a] × 100.

We also defined a positive trend as a value of variation greater than zero and a negative trend as a value of variation lower than zero.

### Statistical Analysis

Statistical analysis was performed with Minitab Software version 20.3 (Minitab LLC, State College, PA, USA). Descriptive statistics are presented as mean and standard deviation, in the case of continuous, normally distributed variables. The median, 25th, and 75th percentiles were used for continuous but non-normally distributed variables. Categorical variables were described in the form of percentages. To decide if the variables used were normally distributed, we used the Ryan Joiner test. We consider that a *p*-value < 0.05 suggests the rejection of the null hypothesis, and thus the values are not normally distributed. To study the relationships between variables, bivariate analysis was used, using the Spearman rho correlation coefficient and quadratic regression. The performed tests were considered statistically relevant if the *p*-value was less than 0.05.

## 3. Results

Twenty-nine patients were included in the study, but during primary data analysis, three patients were excluded because ANC, ALC, and APC were not measured at the specified time points, so twenty-six patients were finally included. The mean age in the study group was 59 (56–70.25) years. Seventeen (65.38%) patients were male, with the rest being female. The mean body max index was 26.2 ± 5.5 kg/m^2^. The mean duration of surgery was 252.4 ± 82.1 min. No patient received a blood transfusion intraoperative or during the first three postoperative days. The median values of the Surgical pleth index were 38. The recorded values intraoperatively for response entropy and state entropy were RE-median values 49.5 Q1:44, Q3:58), and SE-median values 44, Q1:38, Q3:51.

Table 1 shows the general characteristics of the studied group.

The average VO_2_a was 181.0 ± 38.9 mL/min, and the average VO_2_b was 222.3 ± 44.6 mL/min. A positive trend in VO_2_ during general anesthesia has been registered in 84.62% of patients (22 patients), and in four patients (15.38%), VO_2_ was lower at the end of surgery compared to post-induction of anesthesia values. The average neutrophils, lymphocytes, and platelets measured preoperatively and on the third postoperative day are displayed in Table 2.

ANC decreased postoperatively below preoperative values in 11 patients (42.31%), and APC decreased postoperatively in 21 patients (80.77%). ALC showed a negative trend in 24 patients (92.31%). Table 3 presents the variation trend in oxygen consumption, neutrophils, lymphocytes, and platelets.

Using the formulas mentioned above, we calculated the variation of VO_2_, the variation of neutrophil count (VNC), the variation of lymphocyte count (VLC), and the variation of platelet count (VPC).

**Figure 1:**
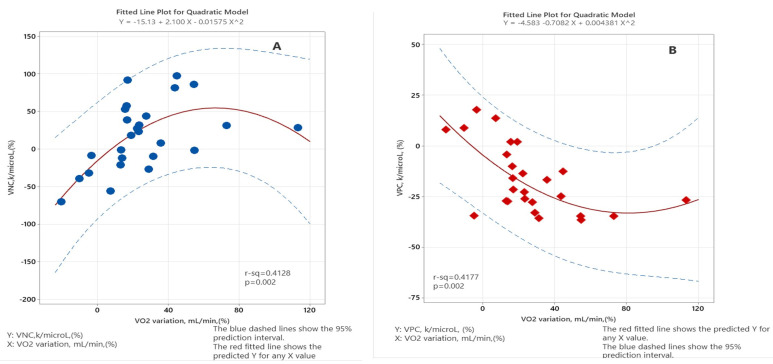
The correlation between VO_2_ variation and ANC variation (**A**) and APC variation (**B**). Legend: VO_2_—oxygen consumption, VNC—variation in neutrophile count, VPC—variation in platelet count.

We observed a significant correlation between the VO_2_ and ANC variations with a positive correlation coefficient (Spearman coefficient = 0.547, *p* < 0.01, 95% confidence interval (CI) = [0.175, 0.783]) and a negative correlation between the APC and VO_2_ variations (Spearman coefficient = −0.603, *p* < 0.01, 95% CI = [−0.815, −0.248]). When applying quadratic regression, we noticed that the relationship between VNC and VO_2_ variation was statistically significant: r^2^= 0.4128, *p* = 0.002. The exact correlation was statistically significant between VPC and VO_2_ variation: r^2^ = 0.4177, *p* = 0.002. We recorded no significant correlation between ALC and VO_2_ variations (*p* = 0.39). Data are presented in Figure 1 and Table 4.

## 4. Discussion

Surgical trauma triggers a systemic response characterized by neuroendocrine and inflammatory changes, activating both the innate and the acquired immune responses [14]. The first moment following surgical trauma is the innate immune system, which becomes active, as the cells involved do not act against a specific antigen. The main activated cells are granulocytes (especially neutrophils and macrophages) and N killer cells, which are white blood cells migrating in a few minutes to the injury site [15]. The specific immunological response comes into secondary action and is characterized by an increase in the Th2/Th1 lymphocyte ratio. This imbalance in the immune response favors both early postoperative complications and oncogenesis. [10,16]. Leukocytes, especially neutrophils, play a central role in the systemic inflammatory response after surgery [17]. The postoperative concentrations of white blood cells peak at 24–48 h and gradually decrease [16]. Our study used ANC on the third postoperative day and showed that in more than half of the studied subjects (57.69%), the ANC remained elevated at 72 h, similar to findings from Romeo et al.’s study on monocyte and neutrophil activity after minor surgical stress, and Prabhu PS et al.’s study on effects of surgical stress on early nonspecific immune response in children [18,19]. These patients must be carefully monitored because they present a worse prognosis concerning the evolution of the oncological disease [20]. As we excluded in our study patients with previous chemotherapy, hematological malignancy, and patients who did not receive blood products perioperatively, we considered neutrophils a surrogate for surgical stress response proportionally to surgical trauma.

VO_2_ can be measured by several methods, one of them being indirect calorimetry, analyzing gaseous respiratory exchanges, or by reverse Fick equation. Of the two methods, indirect calorimetry demonstrated better reproducibility [21]. Although initially published studies claimed that a fraction of inspired oxygen of 0.7 or 0.8 does not influence VO_2_ measurement, Ferreruela et al. demonstrate that by increasing FiO_2_ the accuracy of VO_2_ measurement decreases, and thus recommend a maximum FiO_2_ use of 0.6. [22,23]. We removed the risk of inadequate measurement using a low FiO_2_ of 0.4 to 0.5. Experimentally, general anesthesia was associated with VO_2_ reduction, attributed to reduced muscle tone, reduced cerebral metabolic rate, and hypothermia, whether intentional (e.g., during cardiopulmonary bypass) or secondary (e.g., because of heat loss mechanisms exacerbated during surgery) [24]. Despite these data, a systematic review and meta-analysis, including studies with measurements of VO_2_ before and after induction of general anesthesia (24 studies with 453 patients published 1969–2000), revealed an elevated risk of bias in most studies. A random-effects meta-analysis estimated the reduction of VO_2_ to −33 (95% CI: −38, −28) mL min^−1^ m^−2^ during general anesthesia [25]. In a prospective observational study, VO_2_ was measured by indirect calorimetry (QuarkRMR) before, during, and after major upper abdominal surgery in 20 ASA II-IV patients. VO_2_ decreased by a mean of −46 (95% CI: −55, −38) mL min^−1^ m^−2^ after induction of GA and increased during surgery [21]. Similarly, our study revealed increased VO_2_ variation in most patients (84.62%).

To date, the severity of surgery has been scored preoperatively according to the name of the surgical procedure in existing prediction models. Furthermore, objective intraoperative data are required for better risk prediction of postoperative complications at the end of surgery [26]. Considering the impact of the immune system on postoperative complications and the outcome of oncological disease, we tried to analyze whether the intraoperative VO_2_ dynamic (mirroring the surgical stress) correlates with the postoperative dynamics of neutrophils (a surrogate for the surgical stress response), lymphocytes, and platelets.

Statistical analysis showed a significant correlation between the perioperative variation of ANC and oxygen consumption (Spearman coefficient rho = 0.547, *p* < 0.004), meaning that there is an increase in intraoperative VO_2_ parallels neutrophils. We also performed a quadratic regression, which confirmed the presence of a significant correlation, although this statistical relationship does not imply a cause–effect relationship between VO_2_ variation and ANC variation. We did not find the same correlation between intraoperative oxygen consumption and lymphocytes’ dynamics (*p* = 0.399). However, we can see that in all patients (92.31%), ALC is much lower on the third postoperative day compared to the first count. This highlights a degree of immunosuppression and is also a negative prognostic factor of the postoperative outcome of the oncological patient, both in the short and long term.

The third variable studied was APC; results obtained suggest a significant negative correlation between the APC evolution during the perioperative period and the intraoperative variation of VO_2_ (Spearman coefficient −0.603, *p* = 0.001). In this case, the Spearman coefficient is negative, which means that when the intraoperative trend of oxygen consumption is positive, we expect the APC value determined on day three to be lower than the APC value measured in the preoperative period. The explanations for the low APC value after surgical intervention are either the decrease through hemodilution or through the platelets’ consumption to achieve physiological hemostasis [27]. However, the low number of platelets must be an alarm signal for clinicians because it suggests the activation and aggregation of a very large number of blood platelets, and as Palumbo et al. demonstrated, activated platelets contribute to the progression and distant appearance of metastases [11]. It should also be mentioned that a large number of platelets in the postoperative period is associated with a negative oncological prognosis due to the mechanism called immune thrombosis [28].

In our study, we have obtained a significant correlation between VO_2_ variation, ANC, and APC variation. Still, future studies are needed to set up a reference value of VO_2_ variation, which will prompt the clinician to undertake specific actions to modulate the immune system.

Nociception receptor activation increases heart rate, peripheral vascular resistance, and, implicitly, blood pressure; these changes imply a higher oxygen consumption. The depth of anesthesia is another parameter that can influence oxygen consumption. To ensure that the analgesia and anesthesia during surgery were adequate, we used the surgical pleth index for an objective assessment of pain intensity and response and state entropy for sedation monitoring. We also maintained a normal intraoperative temperature. Since all factors that could influence intraoperative oxygen consumption were kept within the parameters recommended in the literature, we can say that the VO_2_ dynamic is produced by surgical stress and that correlations between the dynamics of the immune system’s cells and oxygen consumption variation are subject to a reduced risk of bias.

Our study tried to fill the gaps between surgical stress, intraoperative oxygen consumption, inflammation, and consequently postoperative complications. The presented data provides a new concept that intraoperative oxygen consumption monitoring may allow a glimpse of the surgery-related stress response apart from its original role and may represent an early indicator for postoperative immune system changes.

This study has its limitations. The small number of patients admitted to the study represents a disadvantage and considering that it is the first time, as far as the authors know, that the VO_2_ variable is used as a predictive factor for the immune system cells’ dynamics, additional studies and investigations are needed to confirm our findings. Although we found a significant correlation between intraoperative oxygen consumption variation and perioperative absolute count neutrophil and platelet variation, the statistical relationship does not imply a causal relationship between the studied variables in all included cases. We believe that an association between oxygen consumption and neutrophil extracellular traps would have a much greater accuracy on the prognosis of the oncology patient’s evolution. Our purpose is to project other studies on the correlation between specific neutrophil populations, intraoperative oxygen consumption, and surgical stress.

## 5. Conclusions

Intraoperative oxygen consumption variation measured by indirect calorimetry correlates significantly with the perioperative absolute neutrophil count dynamic. Intraoperative increases in oxygen consumption correlate with postoperative decreases in thrombocytes. Intraoperative oxygen consumption measurement can be an early indicator of the postoperative immune response dynamic and for better risk prediction of postoperative complications at the end of surgery. Intraoperative quantification of surgical stress severity using oxygen consumption monitoring would thus be valuable for predicting more accurately postoperative complications and for better intraoperative management to suppress postoperative complications. Future studies need to clarify the possible relationship between intraoperative oxygen consumption correlating with perioperative neutrophils and platelets’ dynamic and oncological outcome in colorectal oncological surgery.

## Figures and Tables

**Table 1:** General characteristics of the group.

Parameters	Values
Demographics	
Age ^a^, years	59 (56–70.25)
Gender	
Men ^b^	17 (65.38%)
Women ^b^	9 (34.61%)
BMI ^c^, kg/m^2^	26.26 ± 5.55
Intraoperative data	
Surgical pleth index ^a^	38 (27–56)
Response entropy ^a^	49.5 (44–58)
State entropy ^a^	44 (38–51)
Heart rate ^a^, bpm	65 (59–79)
Temperature ^a^, Celsius degree	36.1 (35.9–36.2)
Systolic blood pressure ^a^, mmHg	104 (85.75–121)
Diastolic blood pressure ^a^, mmHg	66 (57–75)
Oxygen inspiratory fraction ^a^, (%)	45 (40–50)
Surgery and anesthesia characteristics	
Duration of surgery, minutes ^c^	252.4 ± 82.1
Fentanyl, micrograms/kg ^c^	9.975 ± 3.75
Rocuronium, mg/kg ^c^	1.22 ± 0.39
Lidocaine mg/kg ^c^	6.30 ± 2.05
Ketamine, mg/kg ^c^	1.05 ± 0.34

^a^ Median (interquartile range 25–75th percentile), ^b^ percentages, ^c^ mean± standard deviation.

**Table 2:** The average neutrophils, lymphocytes, and platelets perioperatively.

Parameter	Preoperative Values	Postoperative Third-Day Values
Absolute neutrophils count (ANC)	4.57 ± 1.67 × 10^3^/μL	4.99 ± 1.85 × 10^3^/μL
Absolute lymphocyte count (ALC)	1.72 ± 0.85 × 10^3^/μL	1.14 ± 0.52 × 10^3^/μL
Absolute platelets count (APC)	264.1 ± 80.6 × 10^3^/μL	212.35 ± 46.82 × 10^3^/μL

Values are represented by mean ± standard deviation.

**Table 3:** Trend of perioperative variation in oxygen consumption, neutrophils, lymphocytes, and platelets.

Parameter	Number of Patients (%)
**Oxygen consumption variation**	
Positive trend	22 (84.62%)
Negative trend	4 (16.38%)
**ANC variation**	
Positive trend	15 (57.69%)
Negative trend	11 (42.31%)
**ALC variation**	
Positive trend	2 (7.69%)
Negative trend	24 (92.31%)
**APC variation**	
Positive trend	5 (19.23%)
Negative trend	21 (80.77%)

Values are represented as percentages. APC—absolute platelets count, ALC—absolute lymphocyte count, ANC—absolute neutrophils count.

**Table 4:** Descriptive statistics of variation of VO_2_, VNC, VLC and VPC.

Parameter	Values
Variation of VO_2_	20.62 (13.13; 37.57)
Variation of neutrophils count (VNC)	20.91 (−14.31; 46.22)
Variation of lymphocyte count (VLC)	−34.4 (−50.9; −19.4)
Variation of platelets count (VPC)	−22.18 (−29.05; −2.72)

Values are median and 25th and 75th percentiles. Legend: VO_2_—oxygen consumption, VNC—variation in neutrophile count, VPC—variation in platelet count, VLC—Variation of lymphocytes count.

## Data Availability

The original contributions presented in the study are included in the article further inquiries can be directed to the corresponding author.
